# Supplementary material for: Predicting visual function by interpreting a neuronal wiring diagram
Source: Nature. 2024 Oct 2;634(8032):113–23. doi: 10.1038/s41586-024-07953-5 (PMC11446822; doi:10.1038/s41586-024-07953-5)
Supplement: Supplementary file 6 — CRF and ERF predictions for individual TmY4 and TmY9 cells. Analogous to Supplementary Data 3, but for TmY target types. Shown are the top four monosynaptic pathways, the strongest pathway passing through each of the top ten intermediary types (ranking from Extended Data Fig. 7), and the trisynaptic pathway Tm1–TmY–Dm3–TmY (see the section entitled Prediction of spatial normalization). [file 41586_2024_7953_MOESM6_ESM.zip › DataS4/TmY9qΓèÑ/720575940627645821.pdf]

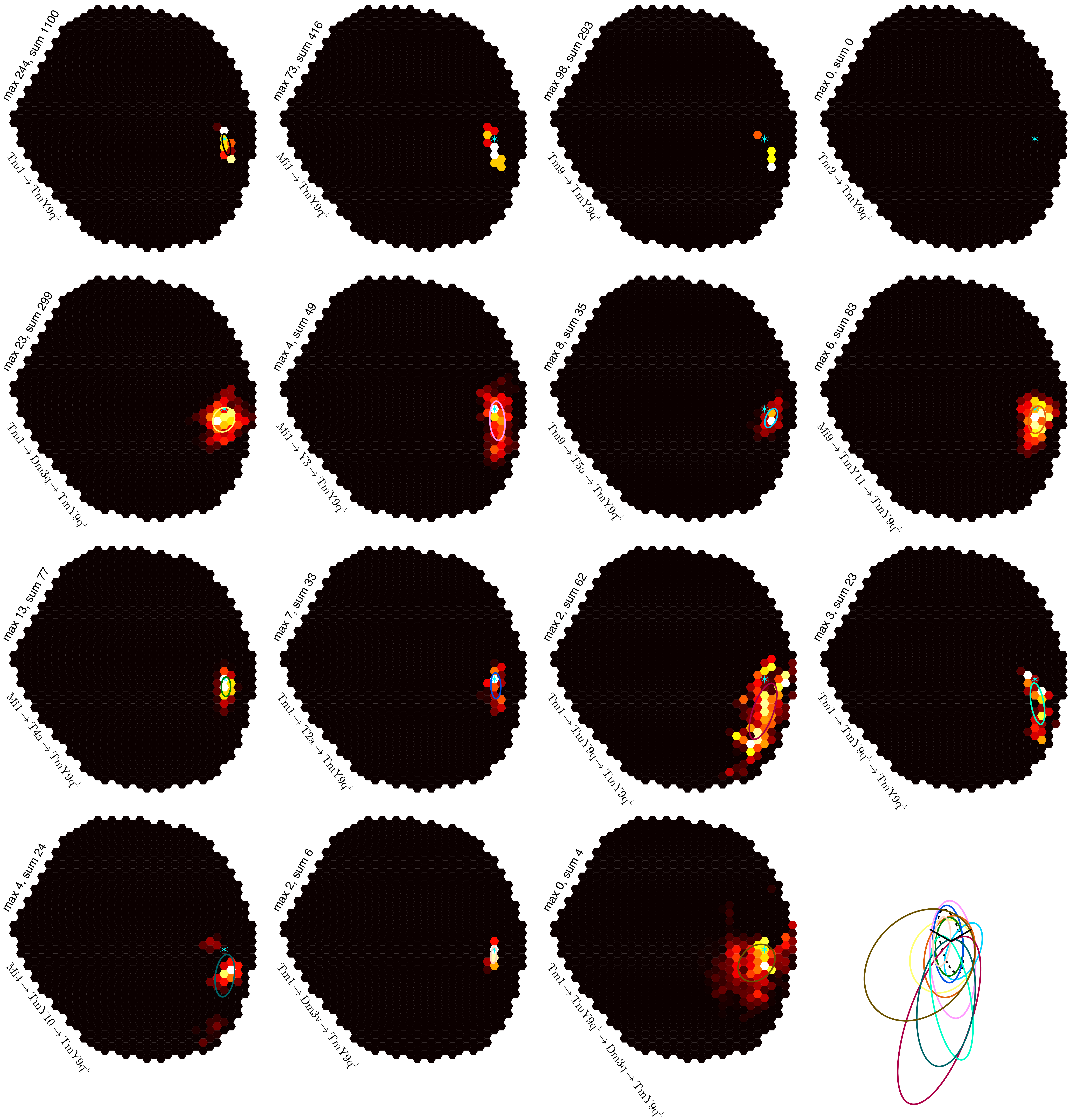

max 244, sum 1100
Tm1 → TmY9q⊥
max 73, sum 416
Mi1 → TmY9q⊥
max 98, sum 293
Tm9 → TmY9q⊥
max 0, sum 0
Tm2 → TmY9q⊥
max 23, sum 299
Tm1 → Dm3q → TmY9q⊥
max 4, sum 49
Mi1 → Y3 → TmY9q⊥
max 8, sum 35
Tm9 → T5a → TmY9q⊥
max 6, sum 83
Mi9 → TmY11 → TmY9q⊥
max 13, sum 77
Mi1 → T4a → TmY9q⊥
max 7, sum 33
Tm1 → T2a → TmY9q⊥
max 2, sum 62
Tm1 → TmY9q → TmY9q⊥
max 3, sum 23
Tm1 → TmY9q⊥ → TmY9q⊥
max 4, sum 24
Mi4 → TmY10 → TmY9q⊥
max 2, sum 6
Tm1 → Dm3v → TmY9q⊥
max 0, sum 4
Tm1 → TmY9q⊥ → Dm3q → TmY9q⊥
